# The Phonograms’ *Genuine‐Character Status*: What the Embedded Semantic Radicals’ Semantic Activation Live by

**DOI:** 10.1002/brb3.70277

**Published:** 2025-01-20

**Authors:** Meng Jiang, Ya Tan, Xia Wang, Yuli Hao

**Affiliations:** ^1^ College of Language Intelligence (College of General Education) Sichuan International Studies University / Chongqing Shapingba District International Joint Institute of Brain Computer Language Interface Chongqing China; ^2^ School of English Studies Sichuan International Studies University Chongqing China

**Keywords:** Chinese pseudo‐character, genuine‐character status‐dependent, semantic activation, semantic radical

## Abstract

**Background:**

In Chinese phonogram processing studies, it is widely accepted that both character and non‐character semantic radicals could be semantically activated. However, little attention was paid to the underlying workings that enabled the semantic radicals’ semantic activation.

**Purpose:**

The present study aimed to address the above issue by conducting two experiments.

**Methods:**

Experiment 1 was committed to confirming whether both character and non‐character semantic radicals could be semantically activated when embedded in genuine Chinese phonograms. Experiment 2 was devoted to exploring whether the same semantic radicals could also be semantically activated when incorporated in Chinese pseudo‐characters.

**Results:**

Results demonstrated that both character and non‐character semantic radicals embedded in the genuine phonograms were semantically activated, but those placed in the pseudo‐characters underwent no semantic activation, suggesting that the semantic activation of semantic radicals was *genuine‐character status*‐dependent, irrespective of the semantic radicals’ characterhood.

**Conclusion:**

It seems that the *genuine‐character status* and the meaning of the host phonogram have strong sway on the semantic activation of semantic radicals.

## 1 Introduction

Chinese, differing sharply from alphabetic writing systems like English, is predominantly an ideographic language. Chinese characters, the basic writing and perceptual unit in the language (Q. Zhang, Zhang, and Kong 2009), are mostly pictophonetic compounds or phonograms. According to Luo, Li, and Zhang (2013), the proportion of phonograms can be as high as 95%. Typically, a phonogram is composed of two types of sublexical units, namely, semantic radical and phonetic radical. Semantic radical usually provides cues for the meaning of the host phonogram, while phonetic radical offers hints or clues for the whole character's pronunciation (Y. Zhou 1978). For example, the phonogram “踩” (/cai3/, to stamp) is composed of the semantic radical “
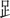
” (/zu2/, foot) and the phonetic radical “采” (/cai3/, to pick). The leftmost “
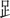
” provides cues for the meaning of the host phonogram which relates to an action executed by the *foot* effector. The rightmost “采” offers a hint for the pronunciation of the whole character.

Based on the semantic relatedness between the semantic radicals and their host phonograms (i.e., *transparency*), phonograms are divided into transparent and opaque ones. Transparent phonograms are those which are semantically bound up with their embedded semantic radicals (e.g., the phonogram “吃” /chi1/, meaning “to eat”). Opaque phonograms, in contrast, bear no semantic relevancy with their embedded semantic radicals (e.g., the phonogram “嗅” /xiu4/, meaning “to sniff”). Depending on the *characterhood* of semantic radicals, they are classified into character semantic radicals and non‐character ones. The former (e.g., the *mouth* radical “口”) can stand alone as an independent character, namely, a simple character, while the latter (e.g., the *hand* radical “扌”) can only combine with other sublexical units to make up a phonogram (Li 2005). Semantic radicals, regardless of their character status, mostly occupy the left side of phonograms, which goes in contrast with phonetic radicals that usually lie on the right side (Feldman and Siok 1999). Approximately 72% of Chinese phonograms are left‐right structured (Hsiao and Shillcock 2006).

### 1.1 Previous Research on the Semantic Activation of Semantic Radicals

So far, a considerable number of studies have been conducted to examine the semantic activation of semantic radicals (X. Zhang et al. 2024), including the modulating variables like semantic radicals’ characterhood and phonograms’ transparency. For example, using a priming lexical decision task, Ding, Peng, and Taft (2004) found that the pre‐exposure of a priming character that served as a sublexical radical of another target character facilitated the processing of the target, demonstrating the activation of the character semantic radical. In a similar vein, Chen and Zhang (2008, 2012) reported the semantic activation of semantic radicals in the recognition of phonograms. Likewise, Q. Wu, Chan, et al. (2013) explored the semantic activation of the *hand* semantic radical “扌” with MRI measurement. Greater activity was observed in the area of right medial frontal gyrus when participants read characters embedding “扌” compared to the condition when they read those without “扌,” suggesting the successful semantic activation of this non‐character semantic radical. L. Zhou et al. (2013), by adopting a primed naming task, found that character semantic radicals embedded in low‐frequency phonograms were semantically activated. Using ERPs technique and a lexical decision task, X. Wang et al. (2017) found that compared with the recognition of opaque phonograms, a shorter reaction time, a smaller P200 and a larger N400 were observed for the recognizing of transparent phonograms, indicating that the character semantic radicals incorporated in transparent phonograms were activated more strongly than those incorporated in opaque phonograms. By adopting a radical priming paradigm and an action direction judgment task, B. Wang et al. (2019) investigated the processing of target characters embedding radicals that conveyed body action meaning (e.g., the hand radical “扌” indicated the upward direction). Results showed a facilitation in the congruent condition where the directions conveyed by the priming radical the target character were congruent (e.g., “扌”—“提,” to lift), while no facilitation was observed in the incongruent condition (e.g., “扌”—“捶,” to thump), suggesting that non‐character semantic radicals’ meaning could be semantically activated. H. Wu, Mai, et al. (2013) examined the somatotopic representation in Chinese verbs, particularly comparing verbs with explicit linguistic cues to the arm, leg, mouth effectors (e.g., “打”/da3/, to hit; “跳”/tiao4/, to jump; “喝”/he1/, to drink) against those without such effector cues (e.g., “割”/ge1/, to cut; “骑”/qi2/, to ride; “尝”/chang2/, to taste). Results showed that effector‐cued verbs exhibited an inverse somatotopic pattern and reduced activation in motor areas, highlighting the semantic activation of these effector semantic radicals. Chen and Zhang (2013) explored the semantic processing of Chinese body verbs with embedded semantic radicals representing mouth, eye, hand, and leg, using lexical naming paradigms. The study examined the congruency between the meanings of host verbs and their embedded radicals (e.g., “打” /da3/, to hit) versus incongruent pairs (e.g., “听” /ting1/, to listen). Results showed that significant differences in reaction times were observed for verbs with different effector organs, regardless of congruency, suggesting successful semantic activation of these effector semantic radicals. R. Zhang et al. (2021) examined the neural adaptation of N170 to the sublexical semantic and phonological processing by manipulating the semantic radicals and the semantic relatedness of the four characters that were presented in sequence. The results showed N170's heightened sensitivity to the semantic processing, suggesting that the semantic radicals were semantically activated. With a priming lexical decision task, Tong et al. (2021) investigated the priming effect of character semantic radicals by categorizing the semantic relatedness between the priming semantic radicals and the embedding target phonograms into five levels. Results showed a graded facilitation in phonogram recognition, revealing the semantic activation of character semantic radicals.

In another vein, researchers such as Chung, Tong, and McBride‐Chang (2012) and Lin et al. (2015) explored whether semantic radicals could be activated when they were incorporated in pseudo‐characters. Chung, Tong, and McBride‐Chang (2012) explored processing differences between pseudo‐characters and non‐characters in typically developing children and those with Chinese dyslexia. Pseudo‐characters contained no actual semantic information but had embedded semantic and phonetic radicals in legal positions, whereas non‐characters had the semantic and phonetic radicals in illegal positions. Results showed that typically developing participants exhibited a greater N400 to pseudo‐characters as compared to non‐characters, suggesting that the embedded semantic radicals might be semantically activated. Lin et al. (2015) examined whether the hand radical “扌” and water radical “氵” were activated when they embedded in pseudo‐characters. They hypothesized that if the semantic information of these semantic radicals could be activated, the hand radical “扌” would elicit greater activation of the premotor cortex as compared to the water radical “氵” given its semantic relation to hand actions. However, no such activation difference was observed, suggesting inactivation of semantic radicals in pseudo‐contexts.

As can be seen, the current research's focus is chiefly placed on the presence of the semantic activation of semantic radicals. It is generally found that the semantic information of semantic radicals can be activated regardless of their character status and their embedding phonograms’ transparency; while very limited effort was made to examine the semantic activation of semantic radicals that were incorporated in pseudo‐characters, and the conclusions were controversial. Moreover, little regard is given to the differential machinery underlying the semantic activation process of semantic radicals in the host transparent and opaque phonograms. Concerning semantic radicals in transparent phonograms, the sublexical semantic activation may arise from the recruitment of the sublexical units’ own semantic information as well as the host transparent phonograms’ semantic information. The latter may well lend a hand. As for semantic radicals in opaque phonograms, the sublexical semantic activation event may be purely a matter of their own semantic information. What on earth are the factors that regulate the sublexical semantic activation event? A further wonder is what would happen if the same semantic radicals are placed in Chinese pseudo‐character contexts. Should they still be semantically activated? In short, the semantic activation of semantic radicals is not just a matter of superficial event. There may be a more complicated story behind. Collectively, the present study is exactly devoted to probing deep into the semantic radicals’ semantic event by taking an advantage of Chinese *foot* semantic radical, in both genuine and Chinese pseudo‐character contexts.

### 1.2 The Present Study

Experiment 1, by employing a semantic‐oriented task, aimed to explore whether both character and non‐character *foot* semantic radicals could be semantically activated when embedded in genuine Chinese phonograms, including transparent and opaque ones.

Since few studies have investigated whether the semantic information of semantic radicals incorporated in pseudo‐characters could be activated, and given that the difference between Chinese genuine and pseudo‐characters is that the former contains semantics while the latter inherently lacks semantic information, which renders it inapplicable to the semantic‐oriented task which is available to the former. Experiment 2, by employing a different research design, was intended to examine whether character and non‐character *foot* semantic radicals could be semantically activated when incorporated in Chinese pseudo‐characters, in an effort to capture the machinery governing the sublexical semantic activation of semantic radicals.

## 2 Experiment 1

### 2.1 Method

#### 2.1.1 Participants

G*power 3.1.9.7 (Faul et al. 2009) was used to calculate the sample size in the present experiment. Forty participants (20 males and 20 females; mean age = 22.67 years, SD = 3.54) were recruited from Sichuan International Studies University (SISU). All of them were native Mandarin speakers, right‐handed, and possessed normal or corrected‐to‐normal vision, with no history of psychiatric or neurological disorders. Prior to the experiment, written consent forms were provided, and they received compensation for their participation.

#### 2.1.2 Materials and Design

The experiment employed a 2 (*Prime image*: foot image vs. control image) × 2 (*Semantic radical type*: character foot semantic radical vs. non‐character foot semantic radical) × 2 (*Transparency of target phonograms*: transparent phonograms vs. opaque phonograms) within‐subject design. The prime foot image depicted a human foot, and the control one was made by distorting the foot image through the utilization of the software GIMP, such that it was no longer discernible. By selecting these two priming images, it is possible to examine the activation of the targets.

With respect to the target phonograms, they differed in the type of semantic radicals. The first type was those that contained the *character foot* semantic radicals which either took on the meaning related to human foot effector (e.g., the target transparent phonogram “踢” /ti1/, meaning “to kick,” contains the character *foot* semantic radical “
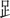
” /zu2/, and denotes the meaning of bodily motion executed by human foot effector), or took on meaning unrelated to human foot effector (e.g., the opaque phonogram “蹭” /ceng4/, meaning “to rub,” denotes foot‐unrelated meaning).

The second type was the same as the first one except that the semantic radicals embedded therein were *non‐character* ones (e.g., the target transparent phonogram “遛” /liu1/, meaning “to walk,” contains the non‐character *foot* semantic radical “辶” /chuo4/, and denotes the meaning of human foot effector‐executed motion; the opaque phonogram “选” /xuan3/, meaning “to select,” denotes no meaning relevant to the bodily motion executed by human foot effector).

An additional type of character was included as controls, which bore a close semantic relationship with human foot effector but did not contain *foot* semantic radicals (e.g., the target character “登” /deng1/, meaning “to step on,” contains no *foot* semantic radical but still denotes the human foot effector‐executed motion meaning). All the materials can be found in Appendix A1.

Of the first two types of Chinese phonograms, each contained 10 transparent and 10 opaque phonograms. Their transparency (i.e., the degree of semantic relatedness between the embedded semantic radicals and the host phonograms) was evaluated on a 7‐point Likert scale (7 = highly related) by 35 participants who were exclusive from the two formal experiments. The rating for the transparent phonograms was significantly higher than that for the opaque ones (*p* < 0.05). The control characters contained 10 characters (see Table 1 for sample materials). The three types of targets were matched in the number of strokes (*F*(2,18) = 2.163, *p* = 0.167), part of speech (both being verbs), and mean frequency (*F*(2,18) = 1.138, *p* = 0.343).

**TABLE 1:** Sample stimuli of primes and targets in Experiment 1.

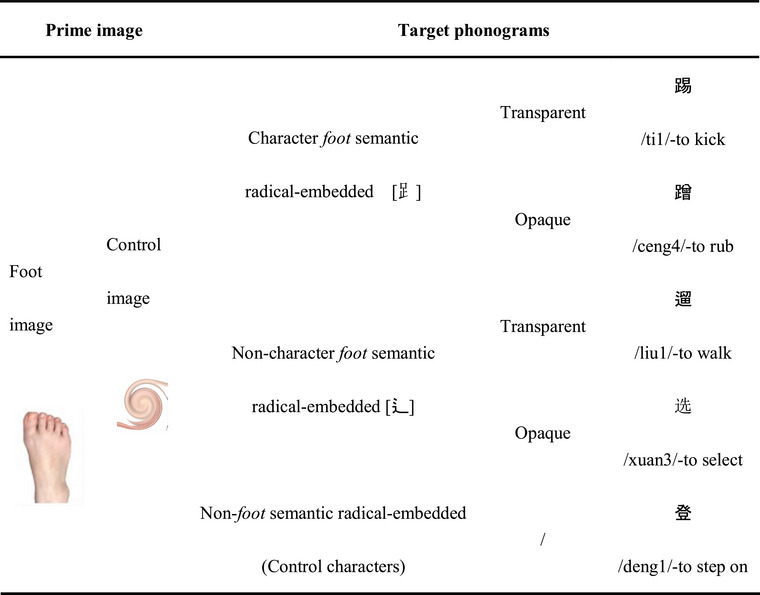

Two types of prime images were matched with 50 target characters and 15 noun controls (for the purpose of meeting the needs of the part‐of‐speech decision task), with a total of 130 prime‐target pairs being fully used across trials.

#### 2.1.3 Procedure

Participants were seated approximately 50 cm from a computer in a comfortable chair. Each trial began with a fixation signal for 300 ms, followed by a blank screen for 300 ms. The prime image was then presented for 500 ms, followed by another 300 ms blank screen. The target (a phonogram, either a verb or a noun) was presented until the participants had made a response. The next trial began after a random interval of 600–800 ms. Participants were instructed to determine whether the target character was a verb or not as quickly as possible by pressing the “F” or “J” keys. The configuration for “pressing key” was counterbalanced across participants, with half using “F” for “NOUN” and “J” for “VERB,” and the other half vice versa. Accuracy and reaction times were recorded. This experiment was conducted in the Key Laboratory of Foreign Language Learning and Cognitive Neuroscience in SISU.

### 2.2 Results

Due to the incomplete data and low accuracy rate (below 90%), data of 31 participants were included in the statistical analysis. The mean accuracy for the primed part‐of‐speech judgment task was 95.01%. The errors and reaction times data exceeding 2.5 SD (5.77%) from the mean RT of each condition were excluded from further analyses. Table 2 exhibits the mean reaction times in Experiment 1. The shortest reaction time (561.19 ms) was observed for the transparent phonograms embedding character *foot* semantic radical in foot image priming condition, then the transparent phonograms embedding non‐character *foot* semantic radical in the same priming condition (604.42 ms) and the opaque phonograms embedding character *foot* semantic radical in foot image priming condition (617.45 ms). The longest reaction time (715.71 ms) was revealed for the opaque phonograms embedding non‐character *foot* semantic radical in control image priming condition.

**TABLE 2:** Mean RT (ms) and SD for part‐of‐speech judgment task in Experiment 1.

Prime image	Semantic radical type	Transparency of target phonograms	
		Transparent phonograms	Opaque phonograms
Foot image	Character *foot* semantic radical [ 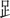 ]	561.19 (83.80)	617.45 (115.55)
	Non‐character *foot* semantic radical [辶]	604.42(87.91)	670.03 (125.26)
Control image	Character *foot* semantic radical [ 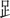 ]	620.78 (198.14)	672.52 (163.20)
	Non‐character *foot* semantic radical [辶]	667.19 (144.45)	715.71 (141.05)

A three‐way repeated measures analysis of variance (ANOVA) was performed on the RTs. Results showed that the main effect of *Prime image* was significant (*F*(1,30) = 11.509, *p* = 0.002, MSE = 16748.754, *η* ^2^ = 0.277). The main effect of *Semantic radical type* was also significant (*F*(1,30) = 38.630, *p* < 0.001, MSE = 3451.091, *η* ^2^ = 0.563). The main effect of *Transparency of target phonograms characters* was also significant (*F*(1,30) = 37.175, *p* < 0.001, MSE = 5146.153, *η* ^2^ = 0.553). The interaction between *Prime image* and *Semantic radical type* was not significant (*F*(1,30) = 0.072, *p* = 0.791, MSE = 2026.256, *η* ^2^ = 0.002). The interaction between *Prime image* and *Transparency of target phonograms* was not significant (*F*(1,30) = 0.782, *p* = 0.358, MSE = 2064.232, *η* ^2^ = 0.028). The interaction between *Semantic radical type* and *Transparency of target phonograms* was not significant (*F*(1,30) = 0.072, *p* = 0.790, MSE = 1983.558, *η* ^2^ = 0.002). The interaction between *Prime image*, *Semantic radical type*, and *Transparency of target phonograms* was not significant (*F*(1,30) = 0.183, *p* = 0.672, MSE = 3394.188, *η* ^2^ = 0.006).

Concerning transparent phonograms, the simple effect analysis showed that the RTs to targets containing the character *foot* semantic radicals preceded by *foot* image primes (561.16 ms) were significantly faster than those preceded by control image primes (620.71 ms) (*p* = 0.002). The RTs to targets containing the non‐character *foot* semantic radicals preceded by *foot* image primes (604.42 ms) were also significantly faster than those preceded by control image primes (667.19 ms) (*p* = 0.010). The difference between the RTs to targets containing non‐*foot* semantic radicals preceded by *foot* image primes (645.16 ms) and to those preceded by control image primes (672.71 ms) were marginally significant (*t*(30) = −1.748, *p* = 0.091).

Two paired samples *t*‐tests were run on the RT differences (i.e., RT_control image_ − RT_foot image_) for the two types of target phonograms. Significantly larger priming effects were found for the targets incorporating the character *foot* semantic radicals (59.52 ms), as compared to the targets incorporating the non‐*foot* semantic radicals (27.55 ms) (*t*(30) = 2.473, *p* = 0.019). Marginally significant larger priming effects were also found for the targets incorporating the non‐character *foot* semantic radicals (62.77 ms), as compared to the targets incorporating the non‐*foot* semantic radicals (27.55 ms) (*t*(30) = 1.991, *p* = 0.056). These results demonstrated that both character and non‐character *foot* semantic radicals embedded in the transparent phonograms were semantically activated.

No significant difference was found in the priming effects between the transparent targets incorporating the character *foot* semantic radicals (59.52 ms) and those incorporating the non‐character *foot* semantic radicals (62.77 ms) (*t*(30) = −0.264, *p* = 0.793). This indicated that the strength of the semantic activation of character *foot* semantic radicals was the same as that of non‐character *foot* ones in the transparent phonograms.

Concerning opaque phonograms, the simple effect analysis showed that the RTs to targets containing the character *foot* semantic radicals preceded by *foot* image primes (617.45 ms) were significantly faster than those preceded by control image primes (672.52 ms) (*p* = 0.027). The RTs to targets containing the non‐character *foot* semantic radicals preceded by *foot* image primes (670.03 ms) were also significantly faster than those preceded by control image primes (715.71 ms) (*p* = 0.002). These results demonstrated that both character and non‐character *foot* semantic radicals embedded in the opaque phonograms were semantically activated.

No significant difference was found in the priming effects between the opaque targets incorporating character *foot* semantic radicals (55.06 ms) and those incorporating non‐character *foot* semantic radicals (45.68 ms) (*t*(30) = 0.401, *p* = 0.691). This indicated that the strength of the semantic activation of character *foot* semantic radicals was the same as that of non‐character *foot* ones in the opaque phonograms.

### 2.3 Discussion

Results from Experiment 1 demonstrated that both character and non‐character *foot* semantic radicals were semantically activated, irrespective of the transparency of the host phonograms. This finding, which goes in alignment with previous studies (e.g., H. Wu, Mai et al. 2013; Tong et al. 2021), falls within our expectation. There is nothing strange that the character *foot* semantic radical could be semantically activated because this type of sublexical unit could copy over its lexical‐level semantic information when used as a standalone character. In other words, the sublexical semantic activation of the character *foot* semantic radical is just a matter of the reproduction of its semantic activation behavior at the lexical level. With respect to the non‐character *foot* semantic radical, it is not in the position to function as an independent character, and thus could afford no such reproduction behavior. However, in Chinese, the non‐character and character *foot* semantic radicals (“辶” and “
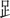
”) often go hand in hand like twin brothers. When in childhood, Chinese native speakers were more than often taught one, seldom in the absence of the other, such that the non‐character *foot* semantic radical “辶” developed a meaning hardly identifiable from that of the character *foot* semantic radical “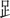.” Given the semantic information acquired when learning the two types of sublexical units, it is not surprising that the non‐character *foot* semantic radical exhibited semantic activation.

Turning an eye on the retrieval of the semantic information of either character or non‐character *foot* semantic radicals, it is noticeable that the host phonograms were all genuine Chinese characters which possessed their own semantic information. A tantalizing question is whether the genuine‐character status of the phonogram and its meaning arising thereof got involved and acted upon the retrieval process. In other terms, it is unclear whether the genuine‐character status and the ensued meaning of the host phonogram constitutes a determinant factor for the *foot* semantic radicals’ semantic activation. Suppose they have played at least a triggering role. In whatever case, it can be said that the semantic activation of the *foot* semantic radical is *genuine‐character status*‐dependent. In simple words, whether the *foot* semantic radical can be semantically activated or not is determined by whether the sub‐lexical unit is placed in a genuine character or not. When placed in a genuine character, it is semantically accessible. When planted in a pseudo‐character context, it may well become semantically inaccessible.

As all the host phonograms recruited in the present study were genuine characters, no definite answer could be provided. A new experiment that employed pseudo‐characters built from the same *foot* semantic radical was desirable. Experiment 2 exactly served this purpose.

## 3 Experiment 2

### 3.1 Method

#### 3.1.1 Participants

Similar to Experiment 1, the sample size was calculated by G*power (3.1.9.7 version), and we adopted the same participants in Experiment 2 as those recruited in Experiment 1.

#### 3.1.2 Materials and Design

Differing from the phonograms in Experiment 1, due to the lack of semantic information in pseudo‐characters, the experiment employed a *2 (Types of semantic radicals: foot vs. control semantic radicals) × 2 (Characterhood of semantic radicals in primes: character vs. non‐character)* within‐subject design.

Primes in this experiment were all pseudo‐characters built by manipulating the types of semantic radicals and the character status of semantic radicals in primes. The two types of semantic radicals were *foot* semantic radicals (i.e., “
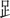
” and “辶”) and control ones (i.e., “耳” and “阝”). The *foot* semantic radicals took on two forms, namely, the character *foot* semantic radical “
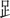
” and the non‐character *foot* semantic radical “辶.” The control (ear) semantic radicals, similarly, also took on two forms, namely, the character *ear* semantic radical “耳” and the non‐character *ear* semantic radical “阝.” All the pseudo‐characters were of the left‐right structures, with all the semantic radicals being placed on the left side. Thirteen simple characters (e.g., “中”, /zhong1/, meaning “middle”) were selected to replace the role of phonetic radicals, such that the combination of the two types of radicals resembled maximally a Chinese real phonogram. In total, there were 4 groups of primes, each of which contained 13 pseudo‐characters (see Table 3 for sample materials, and see Appendix A2 for all the materials).

**TABLE 3:** Sample stimuli of primes and targets in Experiment 2.

Pseudo‐character primes			Targets
Types of semantic radicals	Characterhood	Primes	
*Foot‐related* [ 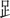 /辶]	Character [ 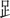 ]	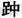	站 /zhan4/‐to stand
	non‐character [辶]	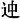	
*Control* [耳/阝]	Character [耳]	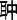	
	non‐character [阝]	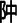	

Thirteen real characters were chosen as targets, which were all semantically related to the *foot* semantic radicals that had been incorporated into the pseudo‐character primes. Besides, all the targets were semantically unrelated to the *control (ear)* semantic radicals that had also been incorporated into the pseudo‐character primes.

As the target characters, which were used for a character decision task, were all genuine Chinese characters, seven pseudo‐characters were created by either adding strokes to or removing strokes from genuine Chinese characters. Four groups of primes were matched with 20 targets (13 genuine characters and 7 pseudo‐characters). In total, 80 prime‐target pairs were utilized across trials.

#### 3.1.3 Procedure

Each trial began with a fixation signal for 300 ms, followed by a blank screen for 300 ms. Then the pseudo‐character prime was presented for 500 ms, followed by another 300 ms blank screen. The target (a character, either a genuine character or a pseudo one) was then presented and remained visible until participants had made a decision. An interval of 600–800 ms was set between the two consecutive trials. Participants were required to decide whether the target character was a genuine character or not as quickly as possible by pressing the “F” or “J” keys. The configuration for “pressing key” was also counterbalanced across participants, with half using “F” for “GENUINE CHARACTER” and “J” for “PSEUDO‐CHARACTER,” and the other half vice versa. Accuracy and reaction times were recorded. This experiment was also conducted in the Key Laboratory of Foreign Language Learning and Cognitive Neuroscience in SISU.

Experiment 2 stood 1 week apart from Experiment 1.

### 3.2 Results

Data of 40 participants were included in the statistical analysis. The mean accuracy for the primed character decision task was 93.65%. The errors and reaction times data exceeding 2.5 SD (6.44%) from the mean RT of each condition were discarded from further analysis. Table 4 exhibits the mean reaction times. The shortest reaction time (525.05 ms) of the targets was observed when the pseudo‐character primes incorporating foot‐related character semantic radicals, then the pseudo‐character primes incorporating foot‐related non‐character semantic radicals (525.32 ms) and the pseudo‐character primes incorporating control character semantic radicals (529.61 ms). The longest reaction time (530.33 ms) was revealed when the pseudo‐character primes incorporating control non‐character semantic radicals (530.33 ms).

**TABLE 4:** Mean RT (ms) and SD for character decision in Experiment 2.

Pseudo‐character primes		Targets
Types of semantic radicals	Characterhood	
*Foot‐related* [ 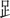 /辶]	Character [ 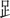 ]	525.05 (74.80)
	non‐character [辶]	525.32 (71.36)
*Control* [耳/阝]	Character [耳]	529.61 (75.51)
	non‐character [阝]	530.33 (73.56)

A two‐way repeated measures ANOVA was conducted on the RTs. Results showed that the main effect of *Types of semantic radicals* was not significant (*F*(1,47) = 0.028, *p* = 0.867, MSE = 416.268, *η* ^2^ = 0.001). The main effect of *Characterhood of semantic radicals in primes* was also not significant (*F*(1,47) = 1.881, *p* = 0.177, MSE = 584.065, *η* ^2^ = 0.038). The interaction between *Types of semantic radicals* and *Characterhood of semantic radicals in primes* was also not significant (*F*(1,47) = 0.005, *p* = 0.944, MSE = 507.043, *η* ^2^ = 0.000). These results indicated that the meaning of semantic radicals embedded in the priming pseudo‐characters was not activated.

The simple effect analysis showed that the RTs to targets preceded by pseudo‐character primes containing the character *foot* semantic radicals (525.05 ms) were not significantly faster than those preceded by pseudo‐character primes containing the character *control (ear)* semantic radicals (529.61 ms) (*p* = 0.301). The RTs to targets preceded by pseudo‐character primes containing the non‐character *foot* semantic radicals (525.32 ms) were not significantly faster than those preceded by pseudo‐character primes containing the non‐character *control (ear)* semantic radicals (530.33 ms) (*p* = 0.335). These results indicated that both character and non‐character *foot* semantic radicals embedded in pseudo‐characters were not semantically activated.

### 3.3 Discussion

The results from Experiment 2 revealed that the semantic information of neither character nor non‐character *foot* semantic radical embedded in the pseudo‐character primes was activated. This, to some degree, went far beyond our expectation. The assumption was that if both the character and non‐character *foot* semantic radical (“
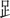
” and “辶”) had developed and possessed their own semantic information, it should have been semantically activated irrespective of whether the sublexical unit was placed in a genuine or pseudo‐Chinese character context. The finding of the present experiment, nevertheless, demonstrated that the *foot* semantic radicals, either character or non‐character ones, once placed in a pseudo‐character context, were no longer accessible. Therefore, it seems that the *genuine‐character status* and the ensued meaning of the host phonogram have played a decisive role in determining whether the semantic information of the *foot* semantic radical is accessible or not. This finding somewhat goes against our intuition. Should the *foot* semantic radical have independent semantic information, it should be retrieved and thus activated free of the influence of the phonogram wherein the sublexical unit is incorporated. However, the results of the present experiment showed that the *genuine‐character status* had indeed involved in and substantially interfered with the *foot* semantic radical's semantic information retrieval. As such, a tentative conclusion may be that the semantic activation of *foot* semantic radical is *genuine‐character status*‐dependent.

Should this conclusion be true, there would arise a couple of very interesting questions: In what ways does the *genuine‐character status* of the host phonogram infiltrate through the accessibility of the *foot* semantic radical? What does the *genuine‐character status* exactly mean? Are there any defining properties that make a Chinese genuine character “genuine” and a pseudo‐character “pseudo”? For a character, does possessing a meaning of its own qualify as this very property? What is the exact property that impacts decisively on the potency of the semantic information of the *foot* semantic radical?… The list of questions can be incessantly added. If the finding of the *foot* semantic radical's *genuine‐character status*‐dependent behavior holds true, a series of mysteries lying underneath would come to the surface and call for unravelling efforts.

In short, the present experiment has produced a very interesting and enlightening finding.

## 4 General Discussion

Experiments 1 and 2 produced contrasting results. Both the character and non‐character *foot* semantic radicals in Experiment 1 were semantically activated. But the same semantic radicals in Experiment 2 failed to undergo semantic activation. These findings, in general, fell out of our expectation. The *foot* semantic radical was possessed of its own meaning whether it was incorporated into a genuine Chinese character or a Chinese pseudo‐character. Should the host phonogram, its *genuine‐character status*, meaning or other properties, have no bother with the *foot* semantic radical's semantic information retrieval, the same sub‐lexical unit in the two different contexts would have been semantically activated in much the same way. But the results were opposite. This suggests that the *genuine‐character status* of the host phonograms had set foot in the semantic radicals’ semantic information retrieval. The point is that the *foot* semantic radical's semantic activation is largely risked upon whether the host phonogram is a genuine character or not. What exactly of the *genuine‐character status* had intervened in this activation event was a tantalizing question, yet to be answered.

In consideration of the Chinese opaque phonograms and pseudo‐characters in Experiments 1 and 2, respectively, which both incorporated the same *foot* semantic radical but differed in that the former had the meaning at the lexical level, while the latter did not, it seemed that the meaning of the host phonogram concerned had played a fate‐deciding role in the *foot* semantic radical's semantic activation. This speculation seemed to be endorsed by the observations of the semantic activation of semantic radicals embedded in genuine phonograms in Experiment 1 of the present study as well as in a big body of previous studies (e.g., X. Wang et al. 2017; Tong et al. 2021), and by the semantic inactivation of semantic radicals in pseudo‐character contexts observed in Experiment 2 and in a few prior studies (e.g., Lin et al. 2015). However, the above finding is based on the examination of the *foot* semantic radical, leaving it uncertain whether other types of semantic radicals would exhibit a similar pattern of behavior or whether alternative experimental paradigms might yield more solid and converging evidence.

Despite the presence of the limitation, this finding is quite novel in relation to previous studies which hardly noticed the *genuine‐character status*‐dependent property of semantic radicals in its semantic activation. Moreover, it may provide ideas and inspirations for the construction of embodied diagnosis and rehabilitation models for the processing disorder of Chinese action verbs embedding effector semantic radicals.

## 5 Conclusion

In Chinese phonogram processing research, it is a general consensus that both character and non‐character semantic radicals can be semantically activated. However, hardly any effort was committed to the underlying workings that enabled the semantic radicals’ semantic activation. The present study, by conducting two experiments, probed into the largely ignored issue. It was found that both character and non‐character *foot* semantic radicals embedded in the genuine character phonograms were semantically activated, but those incorporated in the pseudo‐characters underwent no semantic activation. The tentative conclusion to be arrived at was that the *foot* semantic radical, be it character or non‐character, was *genuine‐character status*‐dependent in its semantic activation. It seemed that the *genuine‐character status* and the meaning of the host phonogram played a decisive role in determining the semantic activation of the *foot* semantic radical. Given that the present study was confined to the *foot* semantic radical, it was not clear whether this conclusion could apply to other types of semantic radicals or not. Future studies would preferably recruit other types of semantic radicals.

## Author Contributions

**Meng Jiang**: conceptualization, funding acquisition, methodology, resources, writing–review and editing. **Ya Tan**: methodology, formal analysis, writing–original draft, writing–review and editing. **Xia Wang**: methodology, formal analysis, supervision, writing–original draft, writing–review and editing. **Yuli Hao**: methodology, data curation, writing–review and editing.

## Ethics Statement

All procedures performed in the study were in accordance with the ethical standards of the institutional and/or national research committee and with the 1964 Helsinki Declaration and its later amendments or comparable ethical standards.

## Consent

Informed consent letters were obtained from all the individual participants included in this study.

## Conflicts of Interest

The authors declare no conflicts of interest.

### Peer Review

The peer review history for this article is available at https://publons.com/publon/10.1002/brb3.70277.

## Data Availability

The datasets and materials generated during the current study are available from the corresponding author on reasonable request.

## 1

Primes and targets used in the experiment.

Table  

**TABLE A1:** Target phonograms and control characters used in Experiment 1.

Item No.	Transparent phonograms embedding the character *foot* semantic radicals	Transparent phonograms embedding the non‐character *foot* semantic radicals	Opaque phonograms embedding the character *foot* semantic radicals	Opaque phonograms embedding the non‐character *foot* semantic radicals	Control characters embedding the non*‐foot* semantic radicals
**1**	踏/ta1/	退/tui4/	踊/yong3/	邀/yao1/	驰/chi2/
**2**	踢/ti1/	遇/yu4/	蹭/ceng4/	递/di4/	骋/cheng3/
**3**	踮/dian3/	避/bi4/	跻/ji1/	遗/yi2/	舞/wu3/
**4**	跺/duo4/	遛/liu1/	跛/bo3/	遮/zhe1/	站/zhan4/
**5**	踹/chuai4/	遁/dun4/	踰/yu2/	逮/dai3/	窜/cuan4/
**6**	跃/yue4/	逛/guang4/	踌/chou2/	逼/bi1/	滚/gun3/
**7**	踩/cai3/	逐/zhu2/	躇/chu2/	造/zao4/	徘/pai2/
**8**	跑/pao3/	遨/ao2/	蹉/cuo1/	逗/dou4/	溜/liu1/
**9**	跳/tiao4/	遣/qian3/	跎/tuo2/	逝/shi4/	登/deng1/
**10**	跨/kua4/	逾/yu2/	趴/pa1/	遍/bian4/	躲/duo3/

**TABLE A2:** Prime pseudo‐characters and targets used in Experiment 2.

## References

[brb370277-bib-0001] Chen, X. , and J. Zhang . 2008. “Role of Familiarity of Semantic Radicals in the Recognition of Highly Familiar Chinese Characters.” Acta Psychologica Sinica 40, no. 2: 149–159.

[brb370277-bib-0002] Chen, X. , and J. Zhang . 2012. “Role of Familiarity of Semantic Radicals in the Recognition of Lowly Familiar Chinese Characters.” Acta Psychologica Sinica 44, no. 7: 882–895.

[brb370277-bib-0003] Chen, X. , and J. Zhang . 2013. “Specific Semantic Processing of Chinese Body Verbs.” Journal of South China Normal University (Social Science Edition) 4: 56–60.

[brb370277-bib-0004] Chung, K. K. H. , X. Tong , and C. McBride‐Chang . 2012. “Evidence for a Deficit in Orthographic Structure Processing in Chinese Developmental Dyslexia: An Event‐Related Potential Study.” Brain Research 1472: 20–31. 22750287 10.1016/j.brainres.2012.06.010

[brb370277-bib-0005] Ding, G. , D. Peng , and M. Taft . 2004. “The Nature of the Mental Representation of Radicals in Chinese: A Priming Study.” Journal of Experimental Psychology: Learning, Memory, and Cognition 30, no. 2: 530. 14979822 10.1037/0278-7393.30.2.530

[brb370277-bib-0006] Faul, F. , E. Erdfelder , A. Buchner , and A.‐G. Lang . 2009. “Statistical Power Analyses Using G* Power 3.1: Tests for Correlation and Regression Analyses.” Behavior Research Methods 41, no. 4: 1149–1160. 19897823 10.3758/BRM.41.4.1149

[brb370277-bib-0007] Feldman, L. B. , and W. W. T. Siok . 1999. “Semantic Radicals Contribute to the Visual Identification of Chinese Characters.” Journal of Memory and Language 40, no. 4: 559–576.

[brb370277-bib-0008] Hsiao, J. H. , and R. Shillcock . 2006. “Analysis of a Chinese Phonetic Compound Database: Implications for Orthographic Processing.” Journal of Psycholinguistic Research 35, no. 5: 405–426. 16897357 10.1007/s10936-006-9022-y

[brb370277-bib-0009] Li, R. 2005. “An Analysis of Meaning Illustration by Pictophonetic Characters Applied in the Teaching of Chinese as a Foreign Language.” Applied Linguistics 2: 104–109.

[brb370277-bib-0010] Lin, N. , X. Wang , Y. Zhao , Y. Liu , X. Li , and Y. Bi . 2015. “Premotor Cortex Activation Elicited During Word Comprehension Relies on Access of Specific Action Concepts.” Journal of Cognitive Neuroscience 27, no. 10: 2051–2062. 26226077 10.1162/jocn_a_00852

[brb370277-bib-0011] Luo, J. , W. Li , and Q. Zhang . 2013. “The Time Course of Chinese Character Recognition: An ERP Evident From a Riddle Guessing Task.” Studies of Psychology and Behavior 11, no. 1: 43.

[brb370277-bib-0012] Tong, X. , M. Xu , J. Zhao , and L. Yu . 2021. “The Graded Priming Effect of Semantic Radical on Chinese Character Recognition.” Frontiers in Psychology 12: 611066. 33708160 10.3389/fpsyg.2021.611066 PMC7940206

[brb370277-bib-0013] Wang, B. , Z. Li , L. Wu , and J. Zhang . 2019. “Effects of Embodied Simulation on Understanding Chinese Body Action Verbs.” Acta Psychologica Sinica 51, no. 12: 1291–1305.

[brb370277-bib-0014] Wang, X. , M. Pei , Y. Wu , and Y. Su . 2017. “Semantic Radicals Contribute More Than Phonetic Radicals to the Recognition of Chinese Phonograms: Behavioral and ERP Evidence in a Factorial Study.” Frontiers in Psychology 8: 2230. 29312076 10.3389/fpsyg.2017.02230 PMC5742193

[brb370277-bib-0015] Wu, Q. , Y. Chan , J. P. Lavallee , H.‐C. Chen , K.‐E. Chang , and Y.‐T. Sung . 2013. “Processing Chinese Hand‐Radicals Activates the Medial Frontal Gyrus.” Neural Regeneration Research 8, no. 20: 1837–1843. 25206492 10.3969/j.issn.1673-5374.2013.20.002 PMC4145974

[brb370277-bib-0016] Wu, H. , X. Mai , H. Tang , Y. Ge , Y.‐J. Luo , and C. Liu . 2013. “Dissociable Somatotopic Representations of Chinese Action Verbs in the Motor and Premotor Cortex.” Scientific Reports 3, no. 1: 2049. 23787364 10.1038/srep02049 PMC6504820

[brb370277-bib-0017] Zhang, X. , W. Cai , M. Dang , R. Zhang , X. Wang , and J. Yang . 2024. “The Neural Correlates of Sub‐Lexical Semantics and Its Integration With the Lexical Meaning in Reading Chinese Characters.” Journal of Neurolinguistics 69: 101176.

[brb370277-bib-0018] Zhang, R. , Z. Wang , X. Wang , and J. Yang . 2021. “N170 Adaptation Effect of the Sub‐Lexical Phonological and Semantic Processing in Chinese Character Reading.” Acta Psychologica Sinica 53, no. 8: 807.

[brb370277-bib-0019] Zhang, Q. , J. X. Zhang , and L. Kong . 2009. “An ERP Study on the Time Course of Phonological and Semantic Activation in Chinese Word Recognition.” International Journal of Psychophysiology 73, no. 3: 235–245. 19358866 10.1016/j.ijpsycho.2009.04.001

[brb370277-bib-0020] Zhou, Y. 1978. “To What Degree Are the ‘Phonetics’ of Present‐Day Chinese Characters Still Phonetic?” [In Chinese.] Zhongguo Yuwen 146: 172–177.

[brb370277-bib-0021] Zhou, L. , G. Peng , H.‐Y. Zheng , I.‐F. Su , and W. S.‐Y. Wang . 2013. “Sub‐Lexical Phonological and Semantic Processing of Semantic Radicals: A Primed Naming Study.” Reading & Writing 26, no. 6: 967–989.

